# Depression as inferential rigidity: a meta-abductive account

**DOI:** 10.3389/fpsyg.2026.1776494

**Published:** 2026-03-03

**Authors:** Jian Sun, Li Jin

**Affiliations:** 1 School of Educational Science, Guangxi Minzu Normal University, Chongzuo, China; 2 School of Philosophy, Zhejiang University, Hangzhou, China

**Keywords:** abduction, cognitive rigidity, depression, inferential pathology, meta-abduction, philosophical psychiatry, rumination

## Abstract

**Introduction:**

Depression is a highly prevalent mental disorder worldwide, and its cognitive rigidity—characterized by persistent negative beliefs resistant to countervailing evidence—remains a critical puzzle in philosophical psychiatry and clinical psychology. Existing theories of abductive reasoning have struggled to explain why similar adversities lead to rigid negative cognition in some individuals but adaptive coping in others.

**Methods:**

Drawing on a hierarchical reconstruction of Peircean abduction, this study develops a three-layer model of depressive inferential pathology. The theoretical framework integrates insights from embodied cognition, existential phenomenology, and epistemic consequentialism to analyze the formal structure of depressive reasoning.

**Results:**

The model identifies three interlocking inferential failures: (1) First-order pathology: Fixation of negatively self-referential abductive explanations, narrowing explanatory space; (2) Core pathology: Failure of meta-abduction, eliminating reflective revision of explanatory practices and cognitive adaptability; (3) Inferential extension: Rigid abductive conclusions are treated as absolute premises for destructive deductive reasoning, generating self-negating conclusions and closed ruminative loops. This model unifies clinical phenomena such as rumination and cognitive distortion, and clarifies the transition from situational responses to chronic pathology.

**Discussion:**

The findings suggest that depressive cognitive rigidity stems not from negative belief content alone, but from structural defects in reasoning. Effective intervention should focus on restoring meta-abductive capacity—treating one’s own explanatory practices as revisable—complementing traditional approaches that target belief correction. This framework bridges philosophical psychiatry and clinical theory, offering a unified account of depression’s cognitive persistence and resistance to intervention.

## 1 Introduction

Depression, as one of the most prevalent mental disorders worldwide (World Health Organization, 2023), has long placed its cognitive–pathological mechanisms at the center of inquiry in philosophical psychiatry and clinical psychology. Although existing theoretical accounts of abductive reasoning are abundant, they struggle to address a crucial question: why do depressive negative beliefs exhibit such remarkable rigidity, remaining resistant even in the face of countervailing evidence? Why do similar life adversities give rise to adaptive coping in some individuals, while leading others into a rigid cycle of negative cognition?

This paper advances a central thesis: a key factor underlying the rigidity and persistence of depressive cognition is a failure of explanatory reasoning—specifically, a failure of abduction at the meta-level. Depressed individuals are not incapable of engaging in explanatory reasoning; rather, they have lost the capacity to reflect upon their own habitual modes of explanation—that is, they suffer from a deficit in meta-abduction. This deficit renders their explanatory chains rigid: first-order abductive reasoning, initiated by unexpected experiences, repeatedly converges on a negative self-conception, thereby forming a closed explanatory loop. The breakdown of meta-abduction prevents the subject from experiencing this rigid chain itself as something surprising, and thus forecloses the possibility of generating alternative explanatory hypotheses. Ultimately, these rigid abductive conclusions are transformed into premises for deductive reasoning, from which conclusions of worthlessness and hopelessness are derived. By shifting the analysis beyond the level of belief content and targeting the formal structure of reasoning, this model offers a novel perspective on the persistence of depressive beliefs and the self-perpetuating nature of depressive cognitive cycles.

The aim of this paper is not to provide a comprehensive etiological theory of depression, but to focus on a frequently neglected yet highly explanatory deficit at the level of reasoning itself. It is crucial to clarify the theoretical scope of our model. We do not posit meta-abductive failure as the singular or sufficient cause of depression, a condition with multifaceted etiologies spanning genetic, neurobiological, and social dimensions. Instead, we propose that once a depressive state is initiated (by whatever combination of factors), the breakdown of meta-abduction functions as a central mechanism that structures and maintains the characteristic cognitive rigidity. Our model aims to elucidate the formal architecture of depressive reasoning—how negative beliefs become entrenched—rather than to provide a comprehensive account of why depression initially emerges. This focus on a maintaining mechanism complements, rather than supplants, other etiological perspectives.

To articulate this central thesis with clarity, two elements of the paper’s theoretical foundation must first be specified. First, the paper adopts an empiricist reconstruction of Peircean abduction. Peirce characterizes abduction as the process of forming explanatory hypotheses in response to a surprising fact (Peirce, 1931–1958). However, the Kantian thesis concerning the unknowability of the thing-in-itself entails that we cannot have direct access to “facts” as such, but only to surprising experiences. Accordingly, the abduction discussed in this paper is understood as a form of explanatory reasoning directed at one’s own subjective experiences. This reconstruction preserves the core logical structure of abduction while rendering it compatible with the phenomenological foundations of research on mental disorders.

Second, the paper introduces a crucial distinction between first-order abduction and meta-abduction. First-order abduction consists in the direct explanation of particular experiences—for example, interpreting a certain inner feeling as “despair.” Meta-abduction, by contrast, is a reflective explanation of one’s own explanatory activity, such as abductively addressing the question of why one interprets a given inner feeling as despair. In essence, meta-abduction is metacognition expressed in abductive form; it constitutes a reasoning-level manifestation of reflective self-examination. The activation of meta-abduction depends on the explanatory activity itself becoming a surprising experience, and it is precisely the failure of this activation mechanism that lies at the core of depressive cognitive pathology.

On the basis of these theoretical commitments, the paper proceeds as follows. Chapter 2 systematically explicates the empiricist content of Peircean abduction and clarifies the hierarchical distinction between first-order abduction and meta-abduction, thereby establishing the theoretical framework for the subsequent pathological analysis. Chapter 3 introduces a three-layer model of depressive inferential pathology, successively examining the formation of negative explanatory chains at the first-order level, the failure of meta-abduction at the core level, and the extended process of destructive deduction. Chapter 4 assesses the explanatory power of this model by using it to unify clinical phenomena such as rumination and cognitive distortion, while also addressing potential objections, including concerns about circularity and excessive intellectualization. Chapter 5 offers the conclusion.

## 2 Theoretical framework: Peircean abduction and levels of reasoning

To understand the pathology of meta-abductive failure in depression, it is first necessary to establish a clear theoretical framework of abductive reasoning. This chapter takes Charles Sanders Peirce’s logic of abduction as its point of departure and offers an empiricist reconstruction of it. The aim is to clarify both the dialogical character of abductive reasoning and its metacognitive basis, while introducing a crucial distinction between first-order abduction and meta-abduction. This distinction constitutes the central theoretical instrument for the pathological analysis developed in the subsequent chapters.

More specifically, the chapter seeks to integrate key insights from existing models of abduction into a unified framework that is suited to analyzing formal pathologies of reasoning. By articulating the hierarchical structure of abductive reasoning and its reflective dimensions, the framework developed here provides the conceptual foundations required for a systematic account of depressive inferential dysfunction.

### 2.1 The core of Peircean abduction and an empiricist reconstruction

At the end of the nineteenth century, Charles Sanders Peirce systematically articulated abduction as one of the three fundamental forms of human reasoning, alongside deduction and induction. In Peirce’s canonical formulation, the logical schema of abduction is as follows: a surprising fact C is observed; if A were true, C would be expected; therefore, there is reason to suppose that A is true (Peirce, 1931–1958). Abduction is a non-demonstrative and ampliative form of reasoning: it does not assign truth to the explanatory hypothesis A, yet it plays a crucial epistemic role by rendering a previously disjointed experience C intelligible and by alleviating the tension generated by surprise. Importantly, abduction is not merely a heuristic for hypothesis generation, but a normative practice whose defeasibility is essential to its rational function. As Reynolds (2017) argues, even ostensibly “non-argumentative” phenomenological methods are guided by abductive anticipation; at their core, *a priori* arguments consist in abductive attempts to identify the best explanation of experiential phenomena.

In this paper, Peirce’s original definition is subjected to an empiricist reconstruction by replacing the notion of a surprising fact with that of a surprising experience. The philosophical motivation for this reconstruction derives from the Kantian epistemological constraint according to which the thing-in-itself (Ding an sich), as mind-independent reality, is not directly accessible to cognition (Kant, 1781/1998). What is directly available to the subject are only experiences as structured by cognitive capacities—whether perceptions of external stimuli (e.g., “the sky suddenly darkens”) or awareness of inner sensations (e.g., “an unprovoked palpitation”). Consequently, the genuine starting point of abductive reasoning is not an objective fact as such, but a cognitive rupture within the stream of experience: when an experience conflicts with established expectations, frameworks, or habits, a sense of surprise arises, and abduction is initiated as a means of restoring intelligibility and order.

This reconstruction does not depart from the core spirit of Peircean abduction; on the contrary, it is more faithful to the logic of inquiry and more consistent with Peirce’s semiotic framework. Peirce famously maintains that abduction is “the only kind of reasoning that can produce new ideas” (Peirce, 1931–1958), and the emergence of new beliefs necessarily originates in moments of puzzlement and surprise within subjective experience. Within Peirce’s triadic semiotics, the interpretant is the cognitive effect a sign produces in an interpreter, constituting an essentially subjective act of understanding. An interpretant can itself function as a subsequent sign-object, thereby generating a chain of unlimited semiosis, which underscores the experiential and subject-relative character of meaning.

Moreover, this reconstruction is particularly well suited to the study of mental disorder. The core of psychopathology in conditions such as depression lies not in systematic misjudgments about objective facts, but in a distortion of the subject’s experiential world (Ratcliffe, 2015). For this reason, taking experience rather than fact as the starting point of abductive reasoning allows for a more precise characterization of cognitive pathology.

### 2.2 The dialogical character of abductive reasoning and its metacognitive basis

Unlike the linear, “inner-monologue” progression characteristic of deductive reasoning, abductive reasoning is essentially dialogical in nature. This dialogicality does not consist in external communication with others, but in an internal cognitive dynamic—namely, the reciprocal questioning and response between first-order cognitive activity and metacognitive activity. Contemporary research on meta-reasoning shows that metacognition is not merely retrospective reflection, but involves the ongoing monitoring and regulation of evidence accumulation and confidence states during reasoning itself (Malaiya and Golden, 2025). We engage in abduction because we initially take some experiential phenomenon to be surprising and in need of explanation, and this reflective awareness of one’s own experience already constitutes the intervention of metacognition. It may thus be understood as a dialogue between the I as cognitive agent and the Me as reflective object. In this respect, metacognition, as the reflective Me, resonates with psychodynamic accounts according to which significant others are internalized as elements of the superego (Blatt and Blass, 1996).

Within abductive reasoning, the role of metacognition manifests at two critical points. First, the generation of surprise depends on metacognitive monitoring: only when the metacognitive system detects a mismatch between a current experience and established cognitive frameworks does the subject experience the sense that “this is puzzling.” Absent such monitoring, an experience may deviate from expectation yet remain unnoticed or tacitly accepted, failing to trigger abduction. Second, the formulation and evaluation of hypotheses depend on metacognitive reflection: posing the question of whether hypothesis A can explain experience C is itself a metacognitive reflection on first-order explanatory activity, while selecting the best explanation among competing hypotheses—inference to the best explanation—constitutes a metacognitive assessment of explanatory adequacy.

From the perspective of cognitive dynamics, the dialogical character of abductive reasoning can be understood as an expression of the creative ordering force described by agapism. According to Peirce, the fundamental motive of cognition is an attraction toward harmony, order, and creativity, a force that manifests cognitively as a movement from disorder to order through symbolic dialogue—from surprise to understanding (Peirce, 1893). The dialogical structure of abduction is precisely the core form of this symbolic dialogue: the interaction between metacognition and first-order cognition drives the generation, testing, and revision of hypotheses, thereby constructing local orders of meaning against the background of an entropic universe. In this respect, abductive cognition closely aligns with Schrödinger’s claim that life feeds on negative entropy (Schrödinger, 1944): cognitive activity, as a higher form of life, exhibits abduction as a concentrated expression of negentropic force.

### 2.3 The distinction between first-order abduction and meta-abduction

On the basis of the dialogical character and metacognitive foundations of abductive reasoning, we can further distinguish two essentially different yet interrelated levels of inference: first-order abduction and meta-abduction. The rationale for this distinction lies in the difference between what is to be explained. First-order abduction targets concrete experiences, whereas meta-abduction takes first-order abductive activity itself as its object of explanation.

#### 2.3.1 First-order abduction: explanatory reasoning about concrete experience

First-order abduction is the most basic form of abduction. Its object is the subject’s concrete experience, whether perceptual or interoceptive. From the explanationalist perspective of the AKM model (Aliseda, 2006; Magnani, 2001), the core task of first-order abduction is to generate a hypothesis such that, if true, it would render a surprising experience intelligible, thereby repairing a cognitive rupture. Its logical structure can be represented as follows:

The subject encounters a surprising experience E (e.g., “chest tightness,” “another person does not respond to a greeting”);

If hypothesis H were true (e.g., “the chest tightness is due to anxiety,” “the lack of response is because the person did not notice”), E would be expected;

Therefore, there is reason to suppose that H is true.

First-order abduction is not confined to explicit causal reasoning, but spans a continuum from perceptual integration to narrative construction. At its most basic level, abduction may take the form of an almost automatic interpretive activity, in which vague, heterogeneous, or as-yet undifferentiated experiential contents are unified into a recognizable and nameable object. As Peirce emphasizes, perceptual judgment is not a purely passive reception, but an abductive process that proceeds without reflective intervention: even in the absence of explicit reasoning, the subject has already synthesized disordered sensations into the determinate experience that “something is thus and so” (Peirce, 1903).

On the basis of such primitive abduction, the subject may engage in further abductive reasoning about the object, explaining its cause, meaning, or responsibility—for instance, interpreting “anxiety” as the result of a work-related failure, or understanding “failure at work” as evidence of insufficient ability. Through incorporating discrete events into causal and meaning relations, such abductions progressively construct temporally extended narrative structures. Regardless of form, their core function is to respond directly to the puzzlement posed by concrete experience, integrating isolated experiential fragments into an existing cognitive framework.

From the action-oriented perspective of the GW model (Gabbay and Woods, 2005), the value of first-order abduction lies not only in explanation but also in action guidance. Interpreting chest tightness as anxiety may prompt relaxation strategies, whereas interpreting it as a cardiac issue may prompt seeking medical care. This tight coupling between explanation and action makes first-order abduction a central cognitive tool in the subject’s interaction with the environment. From this standpoint, the persistence of negative explanations in depression may reflect the fact that such explanations activate energy-conserving behavioral strategies. In extreme circumstances—such as severe abuse or serious illness—such negative interpretations may be contextually appropriate. What renders depression pathological, however, is not the activation of conservative or avoidant strategies as such, but the subject’s failure to recognize the abnormality of the negative explanation itself, and the consequent lack of motivation or capacity to revise it or generate alternatives. This leads directly to the problem of meta-abduction.

#### 2.3.2 Meta-abduction: reflective reasoning about cognitive activity itself

Meta-abduction is a higher-order form of abduction whose object is not concrete experience, but first-order abductive activity itself—that is, explanatory reasoning about why one explains experience E by hypothesis H. Meta-abduction is metacognition in abductive form, a reasoning-level instantiation of reflective self-examination. Its central task is to explain the origin and rational status of cognitive activity, rather than the causes of experience.

Like first-order abduction, meta-abduction requires surprise. However, the object of surprise here is not experience, but the explanatory activity itself. More specifically, when the metacognitive system detects that one’s explanation of experience E by hypothesis H is somehow anomalous—for example, when H conflicts with other evidence, exhibits excessive rigidity, or results in persistent cognitive distress—the subject may experience the puzzlement of “why do I explain things in this way?,” thereby initiating meta-abductive reasoning. Its logical structure can be represented as follows:

The subject becomes aware that they have explained experience E by hypothesis H (a first-order abductive act);
This explanatory activity itself is surprising;
If a meta-hypothesis M were true (e.g., “I explain experiences this way because I possess a negative cognitive schema”), the first-order explanation would be intelligible;
Therefore, there is reason to suppose that M is true.

For example, after experiencing interpersonal rejection, a depressed individual may explain this as others’ dissatisfaction, then further explain that dissatisfaction as evidence of personal defectiveness, which in turn is explained by a supposedly flawed personality, itself traced back to adverse childhood experiences, and so on.

The trigger for meta-abduction is thus the emergence of surprise at the level of one’s own explanatory chain. Once this occurs, the subject is motivated to propose meta-level hypotheses such as “my cognitive schema is negative, which is why I form such abductive chains.” At this point, the hypothesis of a negative cognitive schema acquires plausibility and becomes a proper object of reflection. Indeed, reflective scrutiny of hypotheses generated by meta-abduction can itself constitute a higher-order instance of meta-abduction, allowing for theoretically unlimited recursion.

The central value of meta-abduction lies in its reflective and creative character. Unlike first-order abduction, which focuses on explaining experience, meta-abduction targets the source and rationality of explanations themselves, and can generate new metacognitive insights—for example, the realization that interpreting failure as personal incompetence is a rigid cognitive habit rather than a natural or inevitable explanation. Such insights enable the revision and updating of cognitive frameworks, thereby increasing the flexibility and adaptability of subsequent first-order abductions. From the perspective of epistemic consequentialism (Ahlstrom-Vij and Dunn, 2018), the significance of meta-abduction lies not merely in explaining cognitive activity, but in improving cognitive strategies through reflection, thereby reducing the long-term harms associated with cognitive bias—a hallmark of healthy cognition. One aspect of the depressive predicament, then, may be that negative abductive chains fail to elicit the requisite surprise, preventing the initiation of meta-abductive processes.

#### 2.3.3 The interaction between the two levels

First-order abduction and meta-abduction are not independent, but form a dynamic interactive loop. On the one hand, first-order abduction provides the basis for meta-abduction: without concrete first-order explanations, there is nothing for meta-abduction to reflect upon. On the other hand, the results of meta-abduction feed back into first-order abduction. Metacognitive insights generated through meta-abduction can modify the pattern of hypothesis generation at the first-order level. For instance, only when meta-abduction identifies the interpretation of failure as personal incompetence as rooted in a negative cognitive schema can the subject later generate alternative hypotheses—such as “this failure was due to task difficulty”—thereby disrupting a rigid explanatory cycle.

This interactive loop constitutes a defining feature of healthy cognition. First-order abduction responds to concrete experience, while meta-abduction monitors and optimizes first-order abduction; together, they sustain the cognitive system’s dynamic equilibrium and ongoing development. From the perspective of agapism, this loop represents the core mechanism by which cognition resists entropy. The ordering force driven by agapistic attraction manifests not only in first-order abduction’s integration of surprising experience, but more fundamentally in meta-abduction’s capacity to break closed, single-pattern cycles through symbolic dialogue. Rigid cognition may appear stable, but is in fact disordered: it fails to adapt to changing experiential environments and loses the capacity to counter disorder through dynamic adjustment. By contrast, the sustained interaction between first-order and meta-abduction both preserves cognitive equilibrium and promotes continued evolution in response to new experience, constituting a concentrated manifestation of negentropic force at the cognitive level.

### 2.4 Integrating abductive models and locating cognitive functions

The AKM model and the GW model discussed above may appear to diverge: the former emphasizes the explanatory function of abduction, whereas the latter highlights its action-guiding function. Within the hierarchical framework of first-order abduction and meta-abduction, however, this divergence proves to be superficial. At a more fundamental level, abduction is an embodied practical activity, in which explanatory and action-oriented functions are not separable, but constitute two aspects of a single embodied practice. The concrete form and function of abduction depend on the context in which a problem arises. Such contexts are practical activities through which embodied agents are situated within ecological niches and oriented toward their continued existence.

In the context of first-order abduction, the unity of explanation and action-guidance is expressed in the idea that embodied explanation is itself a form of practice. What the AKM model characterizes as the provision of explanations is, in effect, a practical judgment by an embodied agent aimed at reducing uncertainty and maintaining stability within an ecological niche. In the context of meta-abduction, this unity takes the form of reflective explanation as the optimization of practical strategies. What the GW model describes as “opening an inquiry” is, at bottom, a higher-order embodied activity through which agents adjust their practical strategies via metacognitive reflection. Reflecting on why one consistently explains failure as personal incompetence—a meta-abductive explanation—is not an abstract intellectual exercise, but a critical examination of one’s embodied patterns of practice. The ultimate aim of such reflection is to recalibrate future first-order explanations and action strategies, thereby improving the cognitive system’s fit with its ecological niche. This interpretation aligns closely with the embodied cognition thesis that cognition emerges from the interaction between body and environment: the reflective process of meta-abduction is precisely an embodied practice of optimizing interaction patterns in response to ecological feedback, while the GW model’s notion of “ignorance preservation” functions to preserve flexibility in practical strategy adjustment.

On this integrated conception, the core cognitive functions of abduction can be summarized under two headings: meaning construction and cognitive adaptation. The meaning-construction function is realized at the level of first-order abduction. By providing explanations for surprising experiences, first-order abduction incorporates isolated experiential fragments into a coherent cognitive framework, thereby constructing meaning within an otherwise disordered experiential stream—an account fully consistent with the central commitments of the AKM model. The function of cognitive adaptation is realized at the level of meta-abduction. Through reflective evaluation of the rationality of first-order explanations, meta-abduction enables the adjustment of cognitive frameworks and interpretive habits, allowing the cognitive system to adapt to novel experiential environments and avoid rigidity—an aim that is substantively continuous with the GW model’s emphasis on initiating inquiry.

From the perspective of depressive pathology, it is precisely the imbalance between these two functions that proves decisive. In depression, first-order abduction may become trapped in cycles of negative explanation, representing a distortion of the meaning-construction function, while the failure of meta-abduction prevents reflection upon and revision of these distorted interpretive habits, resulting in a loss of cognitive adaptability. The subsequent chapters will examine this pathological process in detail. For present purposes, however, it is crucial to emphasize that the hierarchical distinction between first-order abduction and meta-abduction, together with their respective roles in meaning construction and cognitive adaptation, provides the central theoretical apparatus for understanding the formal inferential pathology of depression. This framework enables analysis to move beyond surface-level accounts of “negative belief content” and instead to investigate the structural organization of cognitive reasoning itself, thereby revealing the nature of depressive cognitive rigidity.

### 2.5 Chapter summary

This chapter has developed a theoretical framework for analyzing depressive pathology grounded in Peircean abduction. First, through an empiricist reconstruction, the starting point of abduction was shifted from “surprising facts” to “surprising experiences,” aligning the framework more closely with the phenomenological foundations of research on mental disorder. Second, the dialogical character and metacognitive basis of abductive reasoning were articulated, establishing metacognition as central to both the initiation of abduction and the evaluation of explanatory hypotheses. Third, a distinction was drawn between first-order abduction, which explains concrete experience, and meta-abduction, which explains first-order cognitive activity itself, and their interaction and core functions were clarified. Finally, insights from the AKM and GW models were integrated through the lens of embodied cognition, yielding a unified account of abduction’s roles in meaning construction and cognitive adaptation.

The principal contribution of this framework lies in extending the analysis of abduction to the metacognitive level through the introduction of the concept of meta-abduction. This extension provides a crucial perspective for understanding both cognitive flexibility and cognitive rigidity. Building on this framework, the next chapter will introduce a three-layer model of depressive inferential pathology, analyzing in detail the negative fixation of first-order abduction, the failure of meta-abduction, and the destructive deductive processes that emerge from their interaction.

## 3 The inferential pathology of depression: a three-layer model

Building on the hierarchical framework of abductive reasoning established in the previous chapter, this chapter introduces a three-layer model of depressive inferential pathology. The central claim of the model is that a fundamental aspect of depressive cognitive pathology is a systematic inferential disorder that unfolds from the negative fixation of first-order abduction, through the failure of meta-abductive monitoring, and culminates in destructive deductive reasoning. These three layers are mutually reinforcing and together constitute the rigid structure of depressive cognition. This rigidity stands in sharp contrast to the dynamic explanatory cycles driven by agapism in healthy cognition and is best understood as a manifestation of the cognitive system’s failure to resist entropy.

### 3.1 First-order pathology: the formation of negatively self-referential explanatory chains

The core of first-order pathology lies in the monopolization of first-order abductive activity by negatively self-referential explanations, resulting in closed, self-reinforcing explanatory chains. The emergence of such chains is not accidental, but reflects a bias toward prediction-error minimization within the depressed cognitive system. Due to a global transformation in the structure of consciousness (Ratcliffe, 2015), negative explanations are preferentially selected insofar as they fit the subject’s depressive experiential framework, leading to a progressive narrowing of explanatory space.

#### 3.1.1 The initiation of the explanatory chain: from experience to negative naming and reasoning

The explanatory chain begins with the subject’s encounter with concrete experience. Such experience may be endogenous, arising from bodily or affective states (e.g., fatigue, chest tightness, low mood), or exogenous, arising from the perception of external stimuli (e.g., another person’s silence, a work-related setback). From the first-person perspective, however, internal and external experiences are equally given as contents of consciousness and differ only in modality: exogenous experiences are mediated by sensory modalities such as vision or audition, whereas endogenous experiences involve awareness of one’s own physiological states. Experience is pre-conceptual and primordial; as a dynamic and temporally structured whole, it constitutes the subject’s mode of being-in-the-world.

The depressed subject’s initial interpretation of this existential situation typically exhibits two salient features.

First, there is a negative bias in explanatory form. Initial explanations may take the form of negatively valenced naming—for example, directly labeling a vague inner sensation as “I am useless” rather than “I am tired”—or of explicitly inferential reasoning directed at self-defect, such as interpreting another’s silence as “they dislike me” rather than “they may be busy.” Such biases generate irrational self-beliefs, and in traditional CBT frameworks, depression is often attributed to the dominance of these irrational beliefs (Beck and Bredemeier, 2016). The present account, however, advances a deeper claim: the problem is not the irrational belief itself, but the absence of meta-abduction directed at the generation of that belief. This absence, in turn, reflects a global distortion in the self–world relation characteristic of depression. Phenomenological analyses emphasize that the depressed world is experienced as “dead” or inert, no longer affording a plurality of action possibilities (Ratcliffe, 2015; Fernandez, 2014). Value properties are suspended or obscured, affordances collapse, and the space of reasons for action contracts to the minimal goals of maintaining the status quo or avoiding further deterioration. Under such conditions, negative explanations appear not only plausible but natural. Since the negative explanatory chain itself generates no sense of surprise, there is no impetus for critical reflection upon it.

Second, there is self-referentiality in explanatory content. Regardless of the objective source of experience, explanations ultimately converge on alleged intrinsic defects of the self. Bodily fatigue is interpreted as a lack of vitality; ordinary workplace errors are interpreted as evidence of incompetence. Such self-referential interpretations cannot be dismantled through logical refutation or pedagogical correction alone, because they are embedded within a deeper framework that shapes the operation of “rationality” itself. This framework collapses all explanatory dimensions into a single, stable, negative proposition about the essence of the self. Concepts such as “ability” or “vitality” do not denote objective entities, but function as constructed ontological commitments. Through abductive reasoning, diverse and context-specific experiences are attributed to a virtual, stable inner property, thereby reifying what are in fact dynamic activities into static essences. Depressive cognitive rigidity may thus lie in the unreflective acceptance of an essentialist narrative framework, within which the self is treated as an entity with fixed attributes and experience functions merely as evidence for or against these attributes.

This account aligns with core claims of predictive processing theory, according to which cognition is primarily top-down: agents generate predictions first and subsequently test, revise, or accept them in light of incoming information. On this view, depressed subjects are governed by a strong prior centered on an “ineffective self.” Any novel experience is forcibly assimilated to this prior model. Rather than challenging the negative and highly generalized self-schema, novel experiences are conscripted as further confirmation of it, resulting in a rigid, self-validating loop.

#### 3.1.2 The reinforcement of the explanatory chain: layered self-validation

Initial negative explanations do not remain isolated, but initiate a cycle of explanation and re-explanation that progressively strengthens the explanatory chain. The core mechanism is that each negative explanation becomes the “experience” to be explained at the next level, while each subsequent explanation deepens negativity and consolidates self-reference, ultimately directing the chain toward a destructive self-conclusion. The structure can be illustrated as follows:

Initial experience: A supervisor points out a minor error at work (exogenous experience).
First-level explanation: “The supervisor pointed out my mistake, which shows that I am careless.”
Second-level explanation: “I am always careless; this is not accidental but reflects a flaw in my character.”
Third-level explanation: “Because my character is flawed, I cannot perform well in any job; I am a worthless employee.”
Terminal explanation: “As a worthless person, I do not deserve employment or recognition; my existence is a burden.”

Although the first explanation is already negatively self-referential, it remains context-bound. By the terminal stage, however, the explanation has escalated to an existential negation of the self. Suicidal motivation is evidently driven by this level of fundamental self-negation (Lo and Cheng, 2024; Verrocchio et al., 2016): if one’s existence is itself a burden, self-elimination appears as a logically natural conclusion.

The reinforcement of the explanatory chain exhibits two defining features. First, irreversibility: once initiated, the chain moves unidirectionally toward negative conclusions and does not spontaneously shift toward neutral or positive interpretations. Second, self-validation: each explanatory layer supplies justificatory support for the preceding one, rendering the entire chain seemingly coherent. Character flaws explain carelessness; worthlessness explains character flaws. This apparent coherence secures the subject’s conviction and blocks the generation of alternative hypotheses. Even when counterevidence is available, it is ignored or distorted rather than used as a basis for critical reassessment.

The terminal state of the chain is fixation: negative explanation becomes the sole mode of interpreting experience, and explanatory space is fully constricted. This fixation is best understood as a manifestation of cognitive entropy. In healthy cognition, abduction driven by agapism continually generates new hypotheses through symbolic dialogue, sustaining order. In depression, by contrast, the explanatory chain resists reflection and revision, and the cognitive system drifts toward a state analogous to thermal death, where the only remaining “order” is rigid negative self-reference. As noted above, this extreme narrowing of explanatory space reflects domination by a strong prior model of the ineffective self. Neutral or positive hypotheses are not merely rejected; they fail to be generated at all, as they fall outside the subject’s experiential world. It is precisely this total fixation of explanatory space that deprives meta-abduction of its triggering condition and renders reflective intervention impossible.

### 3.2 The Core pathology: the failure of meta-abduction

If the fixation of first-order explanatory chains constitutes the surface pathology of depression, then the failure of meta-abduction constitutes its core pathology. As argued above, meta-abduction is a reflective explanation of first-order abductive activity itself, and its activation depends on first-order explanatory activity becoming a surprising experience. The central cognitive deficit in depression lies precisely in the subject’s difficulty in experiencing rigid negative explanatory chains as surprising. As a result, meta-abduction fails to initiate, and the possibility of revising cognitive strategies is ultimately lost.

#### 3.2.1 The loss of the conditions for meta-abductive activation: the absence of surprise

The activation of meta-abduction requires two conditions. First, the metacognitive system must be capable of detecting anomalies in first-order explanatory activity. Second, such anomalies must elicit a sense of surprise—that is, the puzzlement expressed by the question, “Why do I always explain things in this way?” Depressed subjects exhibit deficits at both points.

First, there is a blunting of metacognitive monitoring. In depressive states, the core functions of metacognition—monitoring, evaluating, and regulating cognitive processes—are significantly weakened (Teodoro et al., 2023). With respect to first-order explanatory chains, this blunting manifests as an inability to register the abnormality of consistently selecting negatively self-referential explanations. The subject passively accepts the outputs of first-order explanation without reflecting on the explanatory process itself.

Second, there is a dissolution of surprise. Because the metacognitive system fails to detect the abnormality of the negative explanatory chain, the depressed subject is unable to experience the requisite sense of surprise. In non-depressed cognition, one would reasonably find it puzzling to interpret every failure as evidence of personal incompetence, thereby triggering meta-abduction and generating meta-level explanations such as “my reasoning may be biased.” For the depressed subject, however, explaining failure in terms of incompetence is fully congruent with their prevailing state of consciousness. Indeed, such explanatory activity is itself a necessary expression of the subject’s globally negative experiential orientation in specific contexts (Ratcliffe, 2015). Consequently, no puzzlement arises, and meta-abduction cannot be initiated. This explains why external persuasion so often proves ineffective: the issue is not stubbornness of character, but the fact that well-intentioned advice operates only at a local level and cannot destabilize the strong prior model sustained by the subject’s global state of consciousness.

The absence of surprise may further be understood as an extension of a diminished sense of reality. The “reality” experienced by depressed subjects is a reality filtered through a negative cognitive framework (Gotlib and Joormann, 2010). Since puzzlement can arise only when there is a rupture between a cognitive framework and an explanatory chain, and since the first-order explanatory chain is itself a constitutive part of that framework, no such rupture occurs. Put differently, the depressive state of consciousness is itself internally incoherent, yet within that experiential world, equally incoherent negative inferential chains appear entirely reasonable.

One might object that this account applies not only to depression, but to cognition in general. After all, explanatory chains are always generated within a cognitive framework; if they were not congruent with that framework, they would not be generated at all. On this view, no one should ever be puzzled by their own beliefs, and meta-abductive reflection would seem impossible in principle.

The response offered here is that healthy cognitive systems are characterized by dynamic coherence. Although explanatory activity at a given time may be congruent with an existing framework, that framework remains open to experience and capable of adjustment through ongoing interaction with new evidence. When circumstances change or novel evidence emerges, previously coherent explanations may lose their plausibility, thereby generating puzzlement. This puzzlement, in turn, triggers meta-abduction, prompting reflection upon and revision of explanatory habits. Depressive cognition, by contrast, exhibits static coherence. Its cognitive framework is closed and rigid, preserving internal consistency by filtering or distorting new experience. As a result, even when the external world changes, explanatory chains do not fracture or generate puzzlement. The key trigger for meta-abduction—experiencing one’s own explanatory activity as surprising—therefore fails, and the system remains trapped within a self-validating loop. This mechanism also helps to explain the widespread behavioral inhibition and avoidance observed in depression (Trew, 2011): action would inevitably involve genuine engagement with the external world, generating novel experiences that resist assimilation to the existing negative framework. By contrast, immobility and avoidance minimize such cognitive conflict, thereby preserving static internal coherence.

#### 3.2.2 The consequences of meta-abductive failure: the loss of cognitive adaptation

The central function of meta-abduction is to sustain the ecological adaptability of the cognitive system—that is, to dynamically adjust cognitive frameworks through reflection on first-order explanatory activity, thereby maintaining sensitivity and responsiveness to changing environmental affordances. The failure of meta-abduction therefore entails the loss of this adaptive capacity. The depressed cognitive system can no longer effectively couple with the richness of its environment and instead becomes confined within rigid negative cycles. Meta-abductive failure, as characterized here, should not be understood as a deficit in inferential competence, but as a failure of rational revisability: the subject remains capable of reasoning, yet loses the capacity to treat their own explanatory practices as open to revision.

The consequences of this failure can be summarized under two headings.

First, there is the permanent fixation of explanatory chains. In the absence of meta-abductive reflection, first-order explanatory chains cannot be revised or disrupted and instead continually reinforce themselves. As Gotlib and Joormann (2010) have shown, depressed individuals exhibit overgeneralized autobiographical memory, failing to extract positive information from specific events that might otherwise revise negative explanations. At a formal level, this reflects the inability of meta-abduction to supply alternative perspectives on first-order explanation.

Second, there is an acceleration of cognitive entropy. In healthy cognition, meta-abduction functions as a primary mechanism for resisting cognitive entropy by generating new meta-hypotheses, updating cognitive frameworks, and maintaining dynamic equilibrium. When meta-abduction fails, the cognitive system can no longer generate new order and instead drifts toward increasing rigidity and disorder. This rigidity is closely associated with the depressive experience of inescapability: depressed subjects are unable not only to escape negative affective states, but also to escape negative cognitive patterns (Gotlib and Joormann, 2010; Seligman, 1975), because the failure of meta-abduction deprives them of the cognitive tools required for escape.

### 3.3 Inferential extension: from closed abduction to destructive deduction

The fixation of first-order explanatory chains and the failure of meta-abduction jointly prepare the ground for the third layer of pathology: destructive deduction. Deductive reasoning derives necessary conclusions from given premises, and its logical validity depends on the truth of those premises. Deduction becomes destructive in depression precisely because rigid negative abductive conclusions (e.g., “I am worthless”) are treated as absolutely true premises. When combined with other premises taken to express unquestionable “truths,” they yield conclusions of self-negation and, ultimately, self-destruction.

#### 3.3.1 The logical structure of destructive deduction: from self-negation to self-destructive tendencies

According to Peirce and later accounts of abduction (Peirce, 1931–1958; Magnani, 2001), abductive reasoning cannot yield certainty. At best, it generates tentative explanatory hypotheses (Aliseda, 2006; Psillos, 2011), which must subsequently be subjected to deductive elaboration and inductive testing. In depressive cognition, however, negative abductive conclusions bypass this evaluative cycle. Their internal coherence supplies a sense of truth, and because meta-abduction fails to operate, these hypothetical explanations are treated as absolute truths. Once negative abductive conclusions are elevated to the status of unquestionable premises, depressed subjects combine them with internalized social norms or value judgments—serving as major premises—and derive destructive conclusions through deductive reasoning.

This inferential process typically unfolds in three stages.

Stage one: the fixation of major and minor premises.

The major premises usually take the form of absolutized value judgments, such as “worthless people do not deserve to live” or “those who burden others ought to disappear.” The minor premises consist of fixed negative abductive conclusions, such as “I am worthless” or “I am a burden to my family.” Within depressive cognition, both premises are treated as absolute truths, rendering the deductive outcome necessary.

Stage two: deductive derivation of self-negating conclusions.

From these premises, depressed subjects deduce conclusions such as “I do not deserve to live” or “I ought to disappear.” These conclusions negate the individual’s existential value and constitute the core cognitive source of suicidal ideation.

Stage three: transformation into behavioral dispositions.

Although the deductive conclusion initially takes the form of a proposition, it is subsequently transformed into an action directive—a shift from declarative to imperative form. When this transformation is completed, the likelihood of dangerous behavior, such as self-harm or suicide, increases substantially. Convinced that they do not deserve to live, depressed subjects come to regard self-elimination as a non-negotiable imperative, and suicide appears as the only viable form of relief.

It is crucial to emphasize that this form of deduction is logically valid. In this respect, depressed individuals do not suffer from a defect in logical competence, and clinically they often exhibit intact or near-intact intelligence. Apparent cognitive impairments—such as attentional narrowing or overgeneralization—are secondary effects of a rigid and stalled cognitive framework. Healthy individuals avoid such conclusions not because they possess superior logical abilities, but because they adopt a tentative, provisional, and revisable attitude toward premises, remaining prepared to revise them through meta-abduction. Negative hypotheses are therefore not treated as absolute premises.

#### 3.3.2 The closed nature of deduction: the structure of rumination

Destructive deduction in depression is not a one-off process but typically takes the form of a persistent loop—clinically identified as rumination, defined as repetitive and passive focus on depressive symptoms and their causes, consequences, and meaning (Nolen-Hoeksema, 1991). From the standpoint of inferential form, rumination is a closed deductive cycle: because meta-abduction has failed, the premises on which deduction operates cannot be revised. The depressed subject cannot acquire new evidence to confirm or disconfirm the conclusion and instead repeatedly re-derives it from the same premises, thereby entering an increasingly hopeless loop.

This closed deductive cycle constitutes a repudiation of Peircean agapism and may be understood as an extreme state of cognitive entropy. In healthy cognition, deductive reasoning is open-ended: conclusions guide action, action generates new experience, and new experience initiates further abduction and induction, allowing premises to be revised in a dynamically progressive cycle. In depressive cognition, by contrast, deduction is closed. Action is inhibited by feelings of inescapability and helplessness, preventing the generation of new experience capable of disrupting the cycle. Cognitive resources are instead consumed within the existing framework, further intensifying depressive states.

It is essential to note that the danger of this deductive loop lies not only in the irrevisability of its premises, but also in the possibility that the premises themselves are actively constructed to serve self-destructive conclusions. This reveals a deeper pathology of rumination: it does not merely spin within a fixed framework, but dynamically generates a self-sufficient argument for self-destruction.

The major premises treated as “absolute truths” in the first stage (e.g., “worthless people do not deserve to live”) are not simply internalizations of external moral norms. Rather, they are often abductively constructed to legitimize an already established self-destructive belief. Once the subject arrives, through negative abduction, at the conclusion “I am worthless,” the ruminative process retrospectively generates or selects an ultimate value judgment capable of rationalizing this conclusion as a major premise. In this sense, the major premise is frequently an abductive product designed to confer legitimacy on the minor premise, rather than the starting point of deduction. Its function is to render subsequent self-negation logically necessary.

Rumination thus plays a dual role. It is both the prison of the deductive cycle and the factory of self-destructive premises. The inferential structure is not static, but dynamically self-completing. On the one hand, abduction generates self-destructive premises by retroactively constructing “worthless persons ought to disappear” from the conclusion “I am worthless.” On the other hand, deduction locks in the necessity of self-destruction by deriving it from those premises within a closed loop. Their mutual reinforcement culminates in the third stage described above: the transformation of the proposition “I do not deserve to exist” into the imperative “I must disappear.”

This account explains why logical refutation of the major premise is often ineffective in depression. The premise is not a neutral moral belief, but a logical component specifically tailored to support a particular self-destructive conclusion. Removing it amounts to undermining the foundation of the entire self-destructive argument, provoking resistance from the cognitive system as a whole. Therapeutic intervention should therefore not focus on disputing the content of the premise, but on dismantling the abductive–deductive loop that binds premise and conclusion, and on restoring meta-abductive capacity, such that the subject can once again experience surprise at the question: “Why do I need to construct such premises in order to justify my own destruction?”

### 3.4 Chapter summary

This chapter has advanced a three-layer model of depressive inferential pathology, characterizing a central cognitive maintenance process in depression as a systematic breakdown of reasoning that spans first-order abduction, meta-abduction, and deductive reasoning. The first-order pathology consists in the formation and fixation of negatively self-referential explanatory chains, whose core features are the narrowing of explanatory space and self-reinforcement. The core pathology lies in the failure of meta-abduction, manifested in the absence of surprise and the inability to generate alternative meta-hypotheses, thereby depriving the cognitive system of adaptability. The inferential extension of this pathology takes the form of destructive deduction, in which fixed negative abductive conclusions function as premises from which self-negating conclusions are derived and repeatedly reinforced within a closed ruminative cycle.

The central contribution of this model lies in its shift away from traditional cognitive theories that focus on the content of negative beliefs, toward an analysis of the form of pathological reasoning. On this view, the defining feature of depression is not that subjects entertain negative thoughts, but that they reason in a rigid and unreflective manner. This perspective not only accounts for the persistence of depressive beliefs, but also resonates with phenomenological insights according to which depression involves a transformation in one’s mode of existence. The next chapter will further examine the explanatory power of this model and address potential objections.

## 4 Explanatory power and objections

This chapter assesses the theoretical value of the abductive inferential pathology model of depression along three dimensions. First, the model is used to provide a unified explanation of common depressive cognitive phenomena observed in clinical practice, thereby testing its explanatory adequacy. Second, the chapter demonstrates the model’s internal affinity with existential phenomenological accounts of depression as an existential transformation, thereby strengthening its philosophical grounding. Finally, it addresses major objections—such as concerns about circularity and excessive intellectualization—clarifying the model’s scope of application and theoretical resilience.

### 4.1 Explaining clinical cognitive phenomena: from rumination to cognitive distortion

The three-layer pathological model developed in the previous chapter is not a merely abstract logical construction. Rather, it maps closely onto the actual cognitive manifestations of depression. This section illustrates the model’s explanatory power by examining two paradigmatic cognitive phenomena commonly observed in clinical contexts.

#### 4.1.1 Rumination: a closed deductive loop

Rumination is widely regarded as a key factor in the maintenance and exacerbation of depression (Treynor et al., 2003; Vélez et al., 2024; Kovács et al., 2020). Traditional cognitive theories typically attribute rumination to the reinforcement of negative beliefs, yet they struggle to resolve a central paradox: why are depressed individuals unable to voluntarily terminate a pattern of thinking that is manifestly distressing? The abductive model offers a more fundamental explanation. Rumination is not merely a matter of “thinking too negatively,” but a closed deductive loop generated by the joint operation of first-order abductive fixation and meta-abductive failure.

First, the model identifies the cognitive conditions that allow ruminative loops to arise and persist. The fixation of first-order abduction—for example, the repeated conclusion “I am incompetent”—supplies deduction with premises treated as effectively absolute. The failure of meta-abduction, in turn, renders these premises immune to reflective scrutiny and revision. As a result, rumination does not originate in the negativity of thought content, but in the structural closure of reasoning itself: deduction merely reiterates conclusions within a fixed premise set and is unable to introduce new explanatory possibilities.

Second, the model clarifies that depressive cognition involves a selective impairment rather than a global breakdown of cognitive function. Traditional accounts often attribute the depressed subject’s difficulty in acquiring corrective experiences to a general “cognitive deficit,” but this cannot explain why intellectual performance remains largely intact in domains unrelated to self-evaluation (Gotlib and Joormann, 2010; Gao et al., 2015). The abductive model instead locates the core impairment in a pervasive negative self-schema that constrains the range of admissible premises. In this sense, depressed individuals are not incapable of reasoning; rather, their reasoning is confined within a closed space defined by a negative self-narrative, preventing the formation of open, revisable premises.

Third, the model captures how rumination gives rise to a second-order vicious cycle through mind–body feedback. As a cognitively demanding activity, rumination continuously consumes resources and intensifies fatigue and feelings of inefficacy (Brosschot et al., 2006; Hjartarson et al., 2021). These bodily states are then interpreted, via rigid first-order abduction, as further evidence for self-negating conclusions (e.g., “this proves that I am indeed useless”), thereby reinforcing the original premises. The resulting loop—rumination → exhaustion → negative explanation → premise fixation → intensified rumination—is self-amplifying. This structure not only explains why rumination is difficult to interrupt, but also reveals why it functions as a central engine of depressive self-maintenance.

#### 4.1.2 Cognitive distortion: the narrowing of explanatory space

Within cognitive science and clinical psychology, cognitive distortions are often regarded as central, if not foundational, to the onset and persistence of depression. Classic cognitive models associated with Beck made seminal contributions in this respect (Beck, 1967), while Ellis identified core forms of irrational belief such as absolutist demands, overgeneralization, and catastrophizing (Dryden and Bond, 1994). More recently, philosophers such as Cohen have characterized irrational cognition as an excessive defensive response to uncertainty, resulting in logical fallacies or hasty reasoning (Cohen et al., 2024). Although these approaches have demonstrated clinical efficacy (Fordham et al., 2021), they do not fully explain why depressed individuals systematically generate and rigidly maintain such negative beliefs, nor why purely content-level logical disputation often proves ineffective.

According to the present model, the essence of cognitive distortion lies in a narrowing of the explanatory space at the level of first-order abduction, rooted in the loss of cognitive adaptability caused by meta-abductive failure. This narrowing is not merely a local “bias,” but the outcome of the cognitive system’s failure to resist entropy. Once the countervailing force of meta-abduction is lost, the system drifts toward rigid disorder, and negative explanations emerge as the only stable option.

In healthy cognition, meta-abduction continuously monitors the adequacy of first-order explanations and generates alternative hypotheses when a single explanation becomes dominant, thereby preserving explanatory plurality. When meta-abduction fails, first-order abduction is monopolized by negatively self-referential interpretations. This manifests in several familiar forms.

First, at the level of explanatory breadth, dichotomous or “black-and-white” thinking reflects an extreme constriction of hypothesis-generation space. Faced with complex situations, the abductive process fails to generate intermediate or graded hypotheses and instead collapses explanatory possibilities into mutually exclusive extremes such as “entirely good” or “entirely bad.” This is not merely an error of judgment, but the systematic deprivation of explanatory alternatives within the cognitive system.

Second, at the level of explanatory depth, catastrophizing manifests as a catastrophic leap within the abductive chain. Rather than proceeding through incremental reasoning, the subject moves directly from a local, concrete negative experience to an existentially devastating conclusion, bypassing all intermediate explanatory steps. This reveals a loss of logical constraint in first-order abduction, whose activity is captured by a strong prior model oriented toward self-negation.

Third, along the temporal dimension, overgeneralization reveals how memory and anticipation are structured by a negative self-narrative. This is not a distortion of “objective experience,” but a consequence of the fact that experience is already filtered through an interpretive framework at the moment it is symbolized and encoded in memory. What is retained is never raw, chaotic experience, but experience already shaped by explanatory activity. In depression, events are encoded within a narrative of personal failure and stored as symbols confirming negative self-traits. With respect to the past, so-called selective memory consists in the continual symbolization of experience under a single negative theme at the cognitive front end. With respect to the future, counterevidence is ignored because prospective positive experiences cannot be assimilated into the existing negative symbolic system and are therefore excluded or neutralized. Past and future are thus unified within a closed hermeneutic loop of symbolic interpretation.

### 4.2 Resonance with existential phenomenology

The present model aligns closely with core insights from existential phenomenology concerning depression. From different theoretical perspectives, the two approaches converge on a shared characterization of the phenomenon. Phenomenologists have argued that the core of depression does not consist in low mood or negative beliefs, but in a fundamental transformation of the subject’s relation to the world (Ratcliffe, 2015). The world is experienced as “dead” or inert, no longer affording possibilities for action, and the subject becomes trapped in a pervasive sense of inescapability. This existential transformation is strictly isomorphic to the inferential pathology described by the present model.

First, the experience of a “dead” world corresponds to the narrowing of explanatory space at the level of first-order abduction. Depressed subjects are unable to generate a plurality of explanations from experience; at the cognitive level, the world loses richness and possibility. This loss reflects, in formal terms, the collapse of the creative function of abductive reasoning.

Second, the sense of inescapability corresponds to the joint operation of meta-abductive failure and closed deductive loops. On the one hand, the failure of meta-abduction prevents depressed subjects from recognizing that they are trapped within negative abductive chains, and thus from disrupting them. On the other hand, destructive deduction ensures that once the conclusion “I am worthless” is reached, rumination retroactively generates a value judgment capable of rationalizing that conclusion as a major premise. This premise then supports the conclusion in return, forming a self-serving and internally coherent loop from which the subject is unable to escape.

Finally, the loss of perceived action possibilities corresponds to a breakdown in the coupling between reasoning and action. According to the GW model, a central function of abduction is to provide hypotheses that guide subsequent action. From the perspective of ecological cognition, action itself depends on the perception of environmental controllability. In this sense, abduction does not merely guide action; it can be understood as constitutive of action itself. On this view, depressed subjects differ from healthy agents in two fundamental respects. First, healthy agents act because they expect their actions to bring about predictable and potentially beneficial changes, whereas depressed subjects interpret any action, regardless of its form, as “doomed to fail” or “devoid of meaning.” Second, in healthy cognition, reasoning guides action, and action-generated experience in turn revises reasoning, forming a dynamic and self-correcting loop. In depression, by contrast, reasoning and action become effectively decoupled, resulting in the loss of ecological coordination between cognition and action.

### 4.3 Responding to core objections

This section addresses the two objections most likely to be raised against the present model. By clarifying key concepts and strengthening the underlying argumentative structure, it aims to reinforce the model’s theoretical resilience.

#### 4.3.1 Objection one and its response

One might object that the present model is overly intellectualized, insofar as it neglects emotional and physiological factors and reduces depression to a mere “problem of reasoning.” The core of this objection is that depression is a complex psychophysical condition involving intense emotional suffering (e.g., despair) and physiological symptoms (e.g., fatigue, sleep disturbance). By focusing on rational inference, the model allegedly ignores these central components and falls prey to a form of intellectualism. The response is as follows.

The model does not deny the reality of emotional and physiological factors. Rather, it seeks to articulate their constitutive relation to cognitive pathology. Emotion is not a parallel accompaniment to cognition, nor is physiology an independent causal factor. Instead, emotion and bodily experience are woven together with cognition within a single inferential network of meaning-making and interpretation.

First, emotion is both an output and a fuel of reasoning. The model does not treat emotion as an external disturbance to reasoning, but as a necessary affective manifestation of a particular inferential structure. When a subject arrives, through a rigid abductive chain, at the conclusion “I am essentially incompetent,” the despair they experience is the emotional equivalent of that inferential outcome. Emotion here is not “noise” interfering with reasoning; it is the felt expression of the meaning generated by the reasoning process itself. At the same time, intense negative affect further narrows attention and rigidifies interpretation, thereby amplifying inferential pathology.

Second, physiological experience constitutes the embodied field of reasoning. Bodily symptoms such as fatigue, psychomotor retardation, or pain are often treated in traditional models as the “biological basis” or “accompanying symptoms” of depression. From the perspective of the present model and of embodied cognition, however, these bodily states form the embodied context in which reasoning unfolds. A rigid inferential framework will directly interpret bodily heaviness as evidence for its own conclusions. The body is thus not a passive physiological substrate, but active material that is interpreted, symbolized, and integrated into a negative network of meaning. Physiology and cognition are not opposing ends of a causal chain; they are continuously co-constituted within processes of meaning construction.

Third, the model offers an integrative framework rather than a reductionist explanation. By emphasizing inferential form, the aim is to capture a multidimensional vicious cycle: physiological exhaustion or genetic vulnerability may increase the likelihood that cognition slides into rigidity; initial inferential biases generate negative content and elicit emotional suffering; emotional suffering, in turn, depletes cognitive resources and increases reliance on rigid, automatic inference. This is a system in which form and content, reason and affect, body and meaning mutually interact.

Fourth, formal analysis points toward deeper forms of intervention. The model’s apparently intellectualized character is in fact the source of its clinical relevance. If the core pathology of depression lies in the failure of meta-abduction, then the focus of intervention should shift away from merely “correcting erroneous beliefs” toward cultivating the capacity to experience one’s own explanatory activity as surprising, disrupting self-referential explanatory chains, and reactivating the generative function of abduction. This orientation resonates with the internal logic of contemporary interventions such as mindfulness-based therapies or acceptance and commitment therapy, which can be understood as projects of reconstructing the cognitive ecological niche. By reshaping the individual’s relation to their own thinking, these approaches re-open possibilities for engaging with the world.

Accordingly, the present model does not present a cold logical schema. Rather, it offers a dynamic picture of how cognition, emotion, and embodiment mutually constrain one another in the collapse of meaning—and how they may also be jointly released. The question it seeks to answer is not “which factor is most important in depression,” but “how these factors together constitute a distinctive and painful mode of existence.”

#### 4.3.2 Objection two and its response

A second objection may be formulated as follows. By locating the core pathology of depression in the rigidity of inferential form, the present model appears to conflict with the widely accepted view that situational depression can be rational. According to epistemic consequentialism (Ahlstrom-Vij and Dunn, 2014), depressive responses following extreme trauma may be etiologically rational—that is, understandable and appropriate reactions to horrific circumstances. To classify such depression as an “inferential pathology” might seem to deny the internal rationality of these responses. The response is as follows.

The present model is not opposed to the claim that situational depression can be rational. Rather, the two approaches offer complementary explanations at different levels of analysis, and the present model provides a more fine-grained account of the mechanisms involved, with direct implications for clinical practice.

First, the model draws a distinction between etiological rationality and the health of ongoing cognitive processes. The notion of etiological rationality emphasized by epistemic consequentialism highlights the intelligibility and appropriateness of depressive responses given certain causes. The present model fully accepts this point. After trauma, first-order explanations such as “the world is dangerous” or “I can no longer trust others” may constitute contextually appropriate and reality-congruent initial abductions. However, the rationality of a response’s origin does not entail that the cognitive processes sustaining it remain healthy. On the present account, regardless of how depression is initially triggered, once cognitive processes become rigid, harm ensues. If explanatory chains remain fixed and meta-abductive capacity fails, then—even given an etiologically rational origin—the cognitive system exhibits the inferential pathology characteristic of depression. This conclusion is consistent with epistemic consequentialism’s overall evaluative framework: even when a state is rationally grounded in its causes, the severe cognitive impairments it produces may render it, all things considered, a rationally inferior condition.

Second, the model identifies the critical transition from reasonable response to pathological state. Specifically, it highlights meta-abductive capacity as the key cognitive pivot distinguishing adaptive sadness from pathological depression. Individuals who are able, over time, to activate meta-abduction—for example, by reflecting on questions such as “Is everyone really untrustworthy?” or “Can my distrust be adjusted in certain safe contexts?”—retain the flexibility and openness necessary for cognitive recovery and are therefore more likely to recover naturally from situational depression. By contrast, individuals whose meta-abductive capacity remains suppressed become trapped in self-validating inferential loops, causing an initially reasonable situational response to solidify into chronic depression detached from its original context. At this stage, the irrationality of depression no longer lies in its initial cause, but in the dysfunction of the cognitive mechanisms that maintain it.

Third, the model provides concrete targets for clinical intervention. In cases of situational depression, therapeutic aims should not be limited to processing traumatic memories as causal factors. They must also include the assessment and cultivation of meta-abductive capacity, in order to prevent a reasonable initial response from hardening into a chronic pathological condition. This requires disrupting the chain of trauma → negative explanation → explanatory fixation → functional impairment, with particular emphasis on intervening at the point of explanatory fixation. In doing so, treatment can respect the rationality of the initial response while effectively preventing its transformation into pathological depression.

In sum, the present model does not deny the rationality of situational depression. Rather, by focusing on cognitive processes rather than etiological causes alone, it offers a mechanistic account of how rational responses can evolve into pathological states. In this respect, it complements and integrates with epistemic consequentialism at a deeper theoretical level.

### 4.4 Chapter summary

This chapter has assessed the theoretical value of the abductive inferential pathology model of depression along three dimensions. First, the model provides a unified explanation of clinical cognitive phenomena such as rumination and cognitive distortion, tracing them back to their underlying inferential forms. Second, the model exhibits a strong internal affinity with existential phenomenological accounts of depression as an existential transformation, thereby grounding the analysis in a robust philosophical framework. Third, by addressing two major objections—concerns about excessive intellectualization and alleged conflict with the rationality of situational depression—the chapter has clarified the model’s core concepts and delineated its scope of application and theoretical resilience.

In sum, the present model moves beyond traditional cognitive theories that focus on the surface content of negative beliefs and instead targets the level of inferential form. In doing so, it preserves the practical tractability of cognitive approaches while incorporating phenomenological insights into the structure of existence and integrating the evaluative framework of epistemic consequentialism. The result is an integrative theory that combines clinical explanatory power with philosophical depth.

While the meta-abductive framework is a theoretical account, its dynamics resonate with several well-established empirical constructs in cognitive and clinical psychology, suggesting pathways for future operationalization and testing. The failure of meta-abduction, characterized by an inability to perceive one’s own explanatory habits as surprising or problematic, directly parallels research on deficits in metacognitive monitoring (e.g., the ability to accurately detect errors or conflicts in one’s thinking). This failure manifests behaviorally as a profound lack of cognitive flexibility—the capacity to adapt thinking and behavior to changing goals or environments—which is a documented impairment in depression (García-Fernández et al., 2025). The resulting rigid, self-reinforcing explanatory chains can be understood as a specific mechanistic explanation for the clinical phenomenon of perseverative thinking (rumination and worry), where individuals become stuck in repetitive negative thought patterns. Furthermore, the model’s final stage, where rigid abductive conclusions are treated as fixed premises for destructive deduction, offers a formal reasoning-level description of impaired belief updating—the difficulty in revising prior beliefs in light of new, countervailing evidence, a key feature of depressive cognition (Zabag et al., 2025). By framing these disparate empirical phenomena through the unified lens of abductive hierarchy and its breakdown, our model aims to provide a coherent conceptual architecture that can help integrate future findings and generate novel hypotheses about the cognitive mechanics of depression.

## 5 Conclusion

This paper has argued that a central mechanism sustaining the cognitive pathology of depression involves a breakdown in the form of reasoning, which underlies the persistence of negative beliefs. By developing a hierarchical framework of abductive inference, it has shown how depressive cognition is structured by three interlocking inferential failures: the fixation of negatively self-referential first-order abduction, the failure of meta-abduction as reflective monitoring, and the subsequent emergence of destructive deductive loops. Together, these failures give rise to the rigidity, self-reinforcement, and apparent inevitability characteristic of depressive thinking.

The central contribution of the proposed model is to shift the explanatory focus from belief-level abnormalities to inferential dynamics. Depression, on this account, is not defined by what is thought, but by how explanations are generated, maintained, and insulated from revision. This formal perspective explains the persistence of depressive beliefs, the resistance to counterevidence, and the transition from intelligible situational responses to chronic pathology. It also clarifies why depressive reasoning can remain logically valid while being epistemically and practically destructive.

By integrating insights from Peircean abduction, embodied cognition, existential phenomenology, and epistemic consequentialism, the model offers a unified framework capable of accounting for clinical phenomena such as rumination and cognitive distortion, while remaining philosophically grounded. At the same time, it avoids both reductionism and intellectualism by situating reasoning within a broader ecology of affect, embodiment, and meaning.

Finally, the analysis has practical implications. If the failure of meta-abduction is central to depressive pathology, then therapeutic intervention should not be limited to correcting belief content, but should aim to restore the subject’s capacity to experience their own explanatory practices as revisable. Future work may further investigate how this inferential framework can be operationalized in clinical contexts and how different forms of depression may vary in their abductive profiles.

## Data Availability

The original contributions presented in the study are included in the article/supplementary material, further inquiries can be directed to the corresponding author.
